# Supplementary material for: Uterine NK cells underexpress KIR2DL1/S1 and LILRB1 in reproductive failure
Source: Front Immunol. 2023 Jan 13;13:1108163. doi: 10.3389/fimmu.2022.1108163 (PMC9880428; doi:10.3389/fimmu.2022.1108163)
Supplement: Supplementary file 1 [file Presentation_1.pptx]

## Slide 1
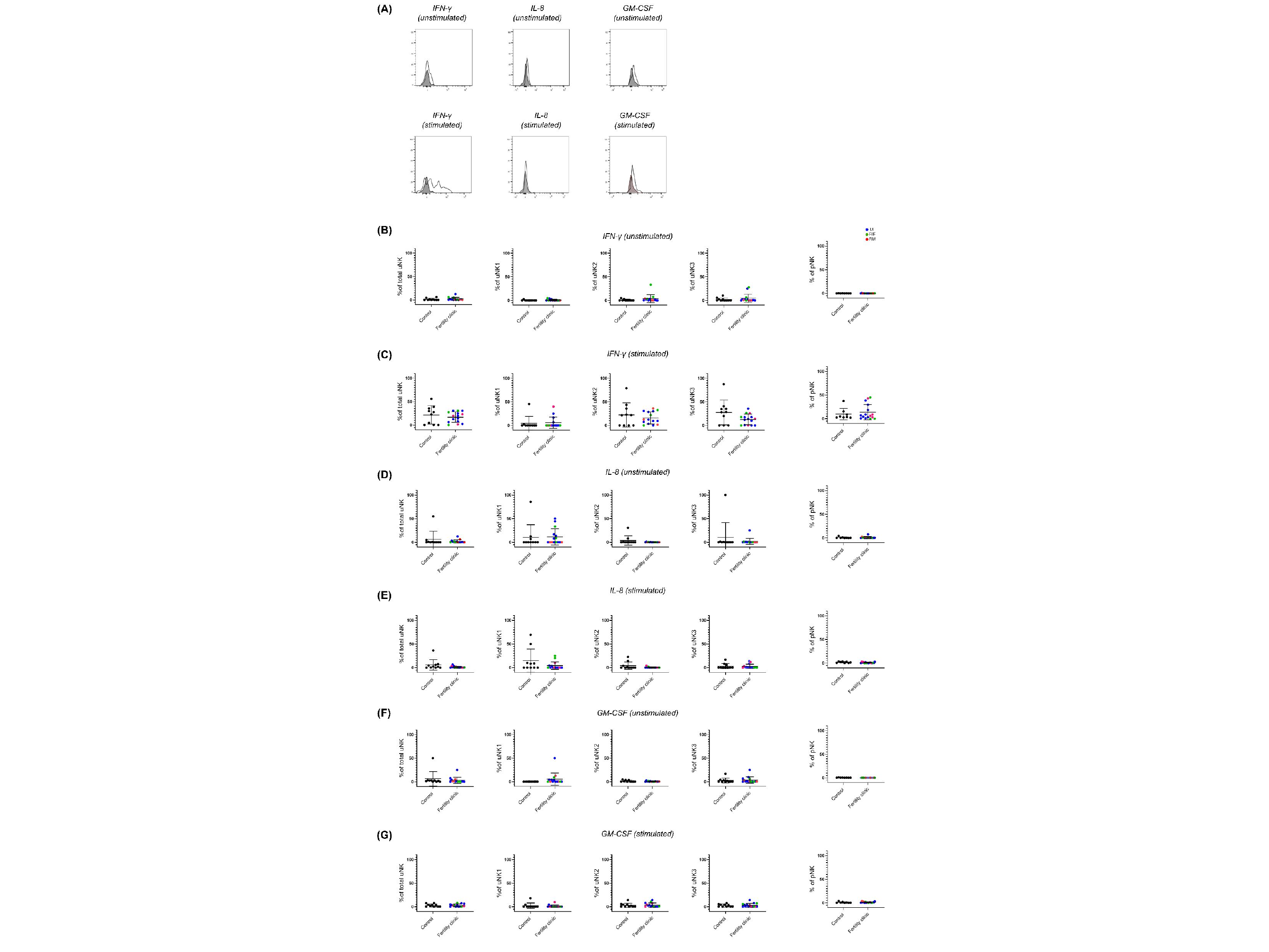

## Slide 2
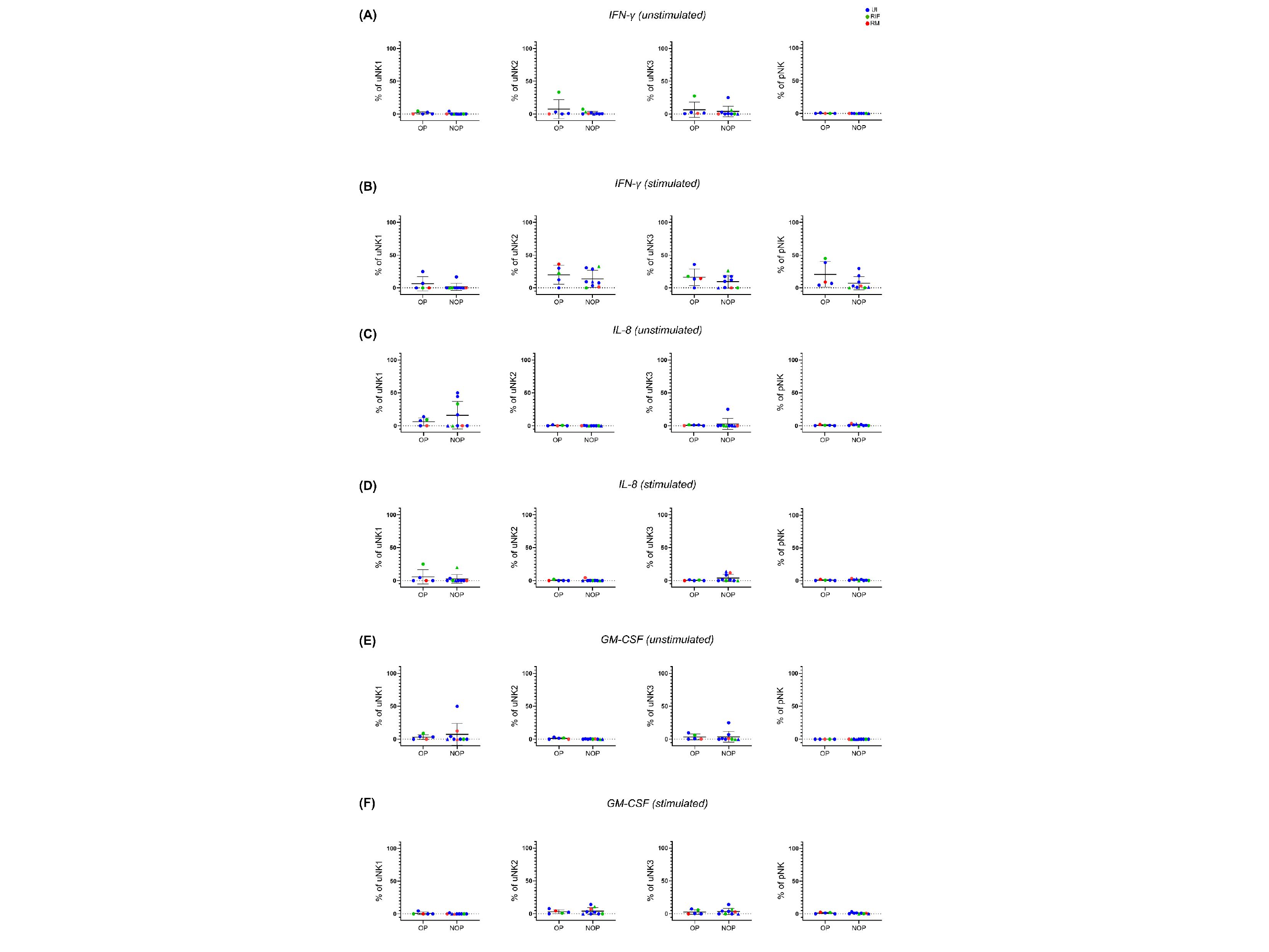

## Slide 3
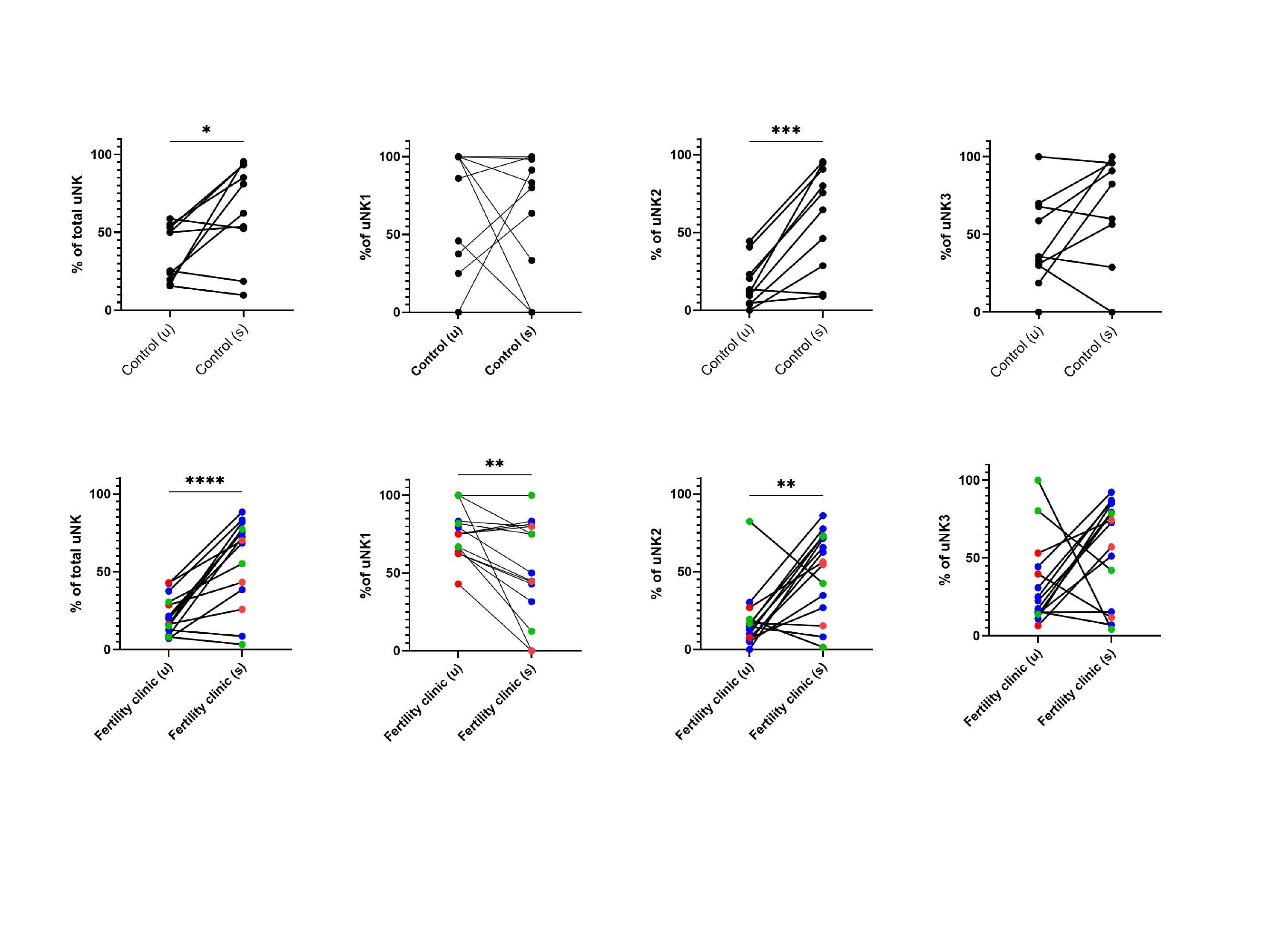

## Slide 4
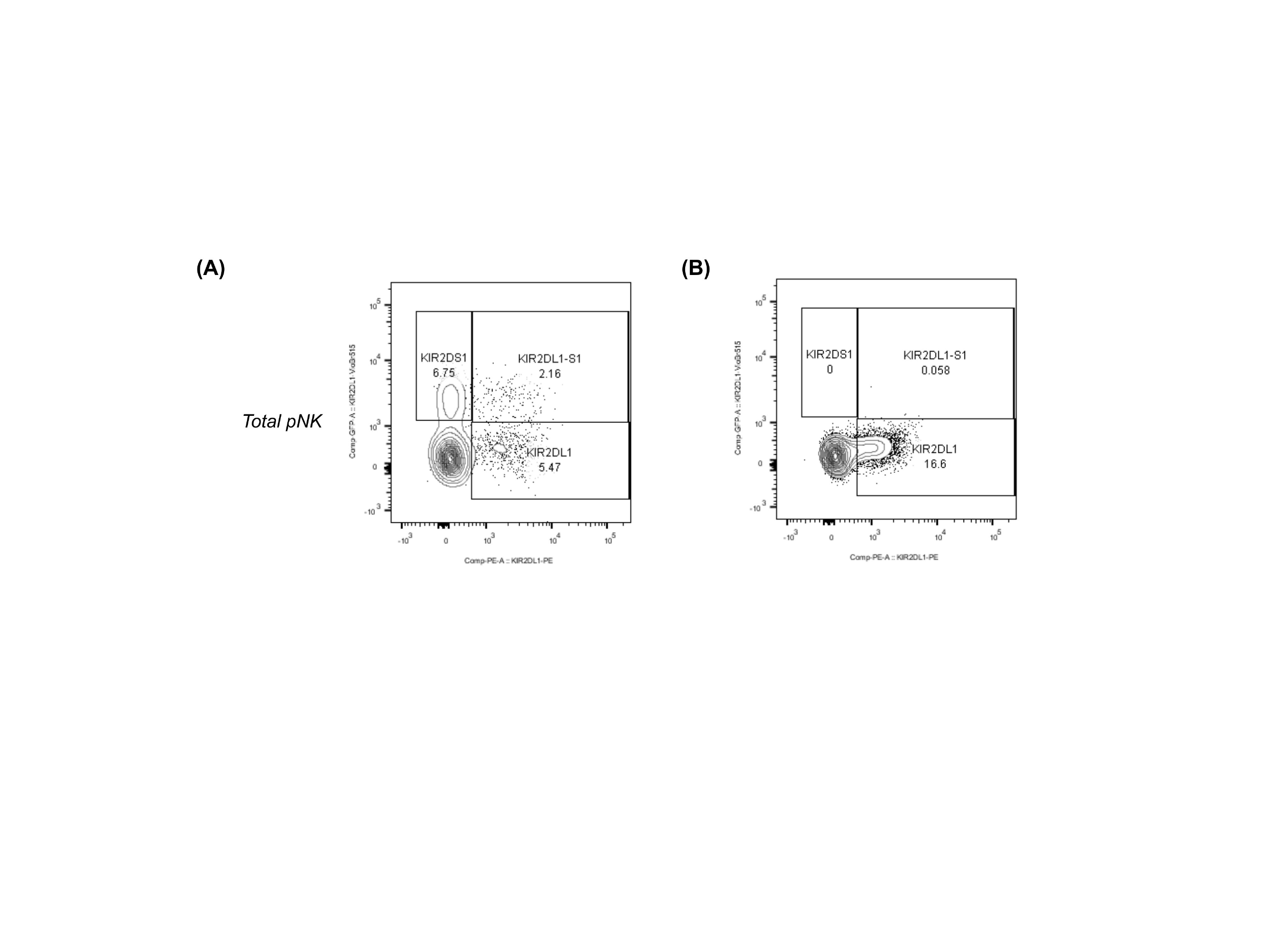

## Slide 5
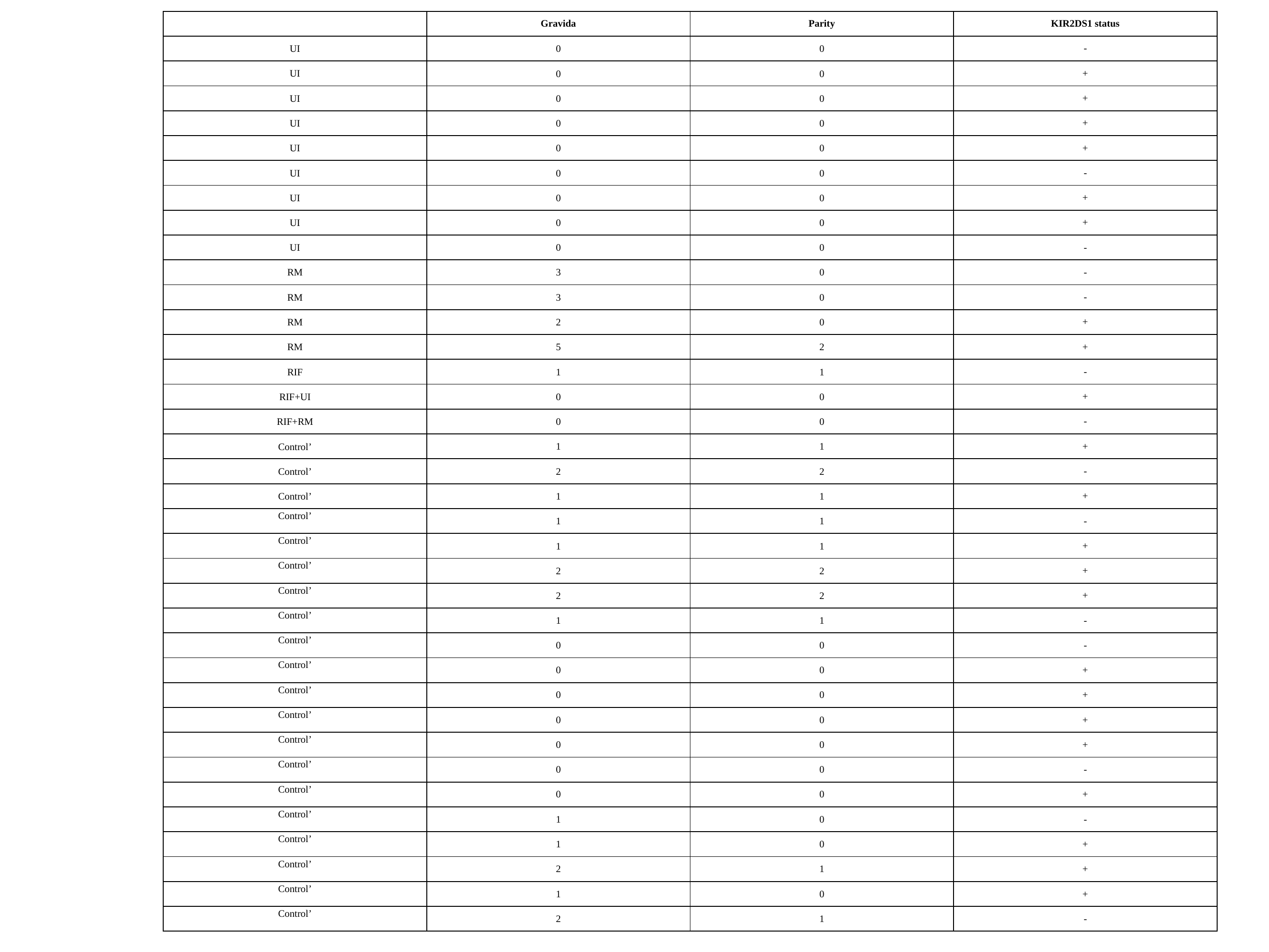

| | Gravida | Parity | KIR2DS1 status |
| --- | --- | --- | --- |
| UI | 0 | 0 | - |
| UI | 0 | 0 | + |
| UI | 0 | 0 | + |
| UI | 0 | 0 | + |
| UI | 0 | 0 | + |
| UI | 0 | 0 | - |
| UI | 0 | 0 | + |
| UI | 0 | 0 | + |
| UI | 0 | 0 | - |
| RM | 3 | 0 | - |
| RM | 3 | 0 | - |
| RM | 2 | 0 | + |
| RM | 5 | 2 | + |
| RIF | 1 | 1 | - |
| RIF+UI | 0 | 0 | + |
| RIF+RM | 0 | 0 | - |
| Control’ | 1 | 1 | + |
| Control’ | 2 | 2 | - |
| Control’ | 1 | 1 | + |
| Control’ | 1 | 1 | - |
| Control’ | 1 | 1 | + |
| Control’ | 2 | 2 | + |
| Control’ | 2 | 2 | + |
| Control’ | 1 | 1 | - |
| Control’ | 0 | 0 | - |
| Control’ | 0 | 0 | + |
| Control’ | 0 | 0 | + |
| Control’ | 0 | 0 | + |
| Control’ | 0 | 0 | + |
| Control’ | 0 | 0 | - |
| Control’ | 0 | 0 | + |
| Control’ | 1 | 0 | - |
| Control’ | 1 | 0 | + |
| Control’ | 2 | 1 | + |
| Control’ | 1 | 0 | + |
| Control’ | 2 | 1 | - |
